# Casirivimab and imdevimab: Cost-effectiveness analysis of the treatment based on monoclonal antibodies on outpatients with Covid-19

**DOI:** 10.1371/journal.pone.0279022

**Published:** 2023-02-10

**Authors:** Matteo Ruggeri, Alessandro Signorini, Silvia Caravaggio

**Affiliations:** 1 Istituto Superiore di Sanità (ISS), Rome, Italy; 2 St. Camillus International University of Health Sciences, Rome, Italy; 3 John Cabot University, Rome, Italy; Universitatsklinikum Schleswig Holstein Campus Lubeck, GERMANY

## Abstract

**Background and objectives:**

In 2020, the world was profoundly affected by the spread of SARS-CoV-2, a novel coronavirus first identified in December 2019, that was the causative agent of coronavirus disease 2019 (Covid-19), a severe respiratory disease classified as a pandemic by the World Health Organization (WHO) in March 2020. Covid-19 had a significant negative impact on the healthcare facilities and the economies of many countries. A need for pharmacological treatments for Covid-19 patients rapidly emerged to limit the damage caused by the disease and allow for more efficient management of hospital resources. A possible alternative treatment that has achieved encouraging results on Covid-19 is the use of monoclonal antibodies. This research aims to evaluate the cost-effectiveness of a type of monoclonal antibody, specifically the combination of casirivimab and imdevimab, and assess its impact on the Italian healthcare system.

**Methods:**

The casirivimab and imdevimab treatment efficacy on outpatients with Covid-19 was tested using a predictive Markov model. Research endpoints include hospitalizations, Intensive Care Unit (ICU) admissions, and deaths. This was translated into terms of benefits (savings) and costs for the Italian National Health Service (NHS). The model operates on a predictive time frame of 20 weeks starting from September 2021 until January 2022. The data used to populate the model comes from international academic studies and open-access resources on online databases.

**Results:**

The model estimates the effects that can be achieved by administering casirivimab and imdevimab treatment on outpatients with Covid-19. According to the estimates, the treatment can prevent approximately 4,000 hospitalizations, 3,589 ICU admissions, and 1,500 deaths in the considered 20-week period. The potential cost savings amount to EUR 78 million, mainly attributable to the reduction in the number of hospitalizations and access to ICU. More specifically, a difference of EUR 15,4 million can be observed due to the reduction in the number of hospitalizations, a difference of EUR 59,3 million due to the reduction in the number in intensive care, and a difference of EUR 20,3 million due to the reduction in deaths as a consequence of the reduction of hospitalizations. These results are already very significant, considering that in Italy, only 4.76% of the population is eligible for monoclonal antibody treatment.

**Conclusion:**

The administration of casirivimab and imdevimab in outpatients with Covid-19 can accelerate recovery from the disease for patients, make hospital resource management more efficient and significantly reduce costs for healthcare facilities.

## 1. Introduction

On March 11^th^, 2020, the World Health Organization (WHO) officially declared a pandemic, caused by the new Coronavirus (SARS-CoV-2) [1], Covid-19 disease. Since then, structural investments in researching new pharmaceutical products for the care of Covid-19 patients have multiplied in quantity and purpose. In Italy, as of October 4^th^, 2021, a total of 4,743,720 cases have been registered [2]. Indirectly, COVID-19 has also caused important economic and health problems. According to a report by GIMBE Foundation [3], only in 2020 severe repercussions on patients and hospitals were experienced due to the missed hospitalizations and medical services in various areas, such as oncology, neurology, and cardiovascular sectors. GIMBE Foundation estimates losses worth more than EUR 5 billion [3].

This state of emergency led the Italian Medicines Agency (AIFA) to approve on February 6^th^, 2021, on a temporary basis, the administration of casirivimab and imdevimab (cas&im) on patients suffering from Covid-19 [4]. Cas&im is a cocktail made up of two noncompeting, neutralizing human IgG1 antibodies that target the receptor-binding domain of the SARS-CoV-2 spike protein and prevent viral entry into human cells through the angiotensin-converting enzyme 2 (ACE2) receptor [5]. In phase III of an outpatient study by Regeneron Pharmaceuticals [6], cas&im proved to significantly reduce the duration of symptoms, hospitalization, ICU, and death rates. By November 2021, cas&im are administered to a limited percentage of patients infected with Covid-19, equal to approximately 4.76% [7].

The drug was approved by the European Medicines Agency (EMA) [8] on November 12^th^, 2021. The recommended posology is a single 600 mg dose of casirivimab and a single 600 mg dose of imdevimab administered intravenously or subcutaneously. The posology differs from the recommendation in the Emergency Use Act where the dosage was a single 1200 mg infusion of casirivimab and a single 1200 mg infusion of imdevimab [9].

The most recent guidelines of Italian Medicines Agency (AIFA), published in the Official Gazette no. 282, November 26^th^, 2021, for the administration of cas&im (Ronapreve), are the following:

- Treatment of COVID-19 in adults and adolescents aged 12 years and older weighing at least 40 kg who require no supplemental oxygen and are at greater risk of progressing to severe COVID-19.
- Prevention of COVID-19 in adults and adolescents aged 12 years and older weighing at least 40 kg

AIFA recommends additional monitoring for the drug. Cas&im should be administered within seven days of onset of symptoms of COVID-19 disease.

This study will only investigate the indication of cas&im for the treatment of COVID-19 in adults and adolescents aged 12 years or older and weighing at least 40 kg, who do not require supplemental oxygen therapy and are at greater risk of progression to severe forms of COVID-19. Thanks to the emergency authorization of AIFA, a monitoring register has been established that allows an estimation of the number of patients eligible for treatment.

## 2. Methods

### 2.1 Structure of the model and hypothesis

**Fig 1:**
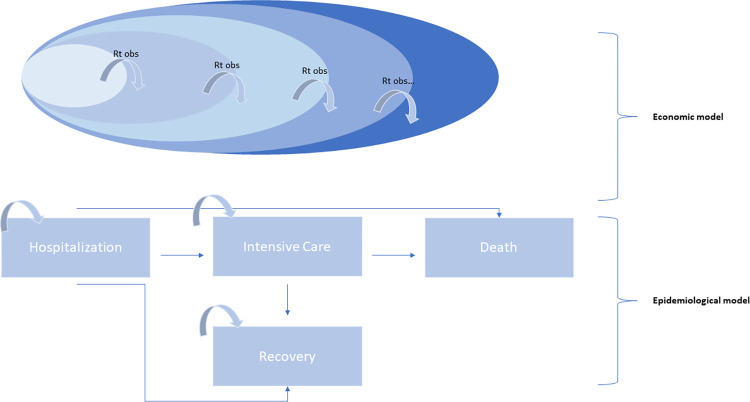
Epidemiological model and economic model. Graphical representation of the model.

The predictive model in this study is based on a model previously adopted by the authors of this report on different economic evaluations [10–12]. The model has been adapted for this research and can be divided into two parts (Fig 1):

1. *Epidemiological Model*: simulates the course of the pandemic in Italy through the simulation of the contagion index (Rt)
2. *Economic Model*: offers an estimate of the costs incurred by the Italian National Health System employing variables such as costs and duration of hospitalizations and intensive care, considering two alternative treatments, standard of care and cas&im treatment, in outpatients with Covid-19

The model operates on a 20 weeks’ timeframe, starting from September 2021. The first phase of the model provides an estimate of the number of Covid-19 positive people, number of hospitalizations, ICU admissions and deaths, on a weekly basis thanks to the Rt index. The next phase consists of the estimation of costs through a Markov model that allows the evaluation in the different states of a patient, in detail, hospitalization, intensive care, recovery, and death, through the relative probabilities of transition.

### 2.2 Population of the model

The data used to populate the model were selected from international clinical studies on the topic of monoclonal antibodies in the case of Covid-19 infection [5, 13] and official Italian and international sources [14–19]. Subsequently, the collected data were shared and validated by Roche Spa’s team of experts.

During the data selection process, assumptions were made due to the lack of important parameters to complete the analysis, in particular, the duration (days) of intensive care and the duration (days) of progress to the state of death. In these cases, it was assumed that, with the cas&im treatment in the worst possible scenario, both the length of stay in ICU and the days of progress to death remain constant.

### 2.3 Clinical data

Details of the value of the parameters in the first simulation phase are shown in Tables 1 and 2. The contagion index (Rt) was estimated with reference to historical data collected from the John Hopkins University open-access database [18] The rates of hospitalization and intensive care admissions were collected from the official Agenas’ website [14], the eligibility rate for treatment was extrapolated from the AIFA report no. 33 [7], while the mortality rate was collected from the Italian National Institute of Health [15]. The percentages of reduction in the risk of hospitalization, ICU admission, and death, resulting from the administration of cas&im, were extrapolated from Weinreich et al. [5].

**Table 1:** Clinical and epidemiological data. Data used to populate the first part of the model.

Parameter	Value	Source
**Rt week 1**	1.10	[18]
**Rt week 2**	1.03	[18]
**Rt week 3**	1.01	[18]
**Rt week 4**	0.95	[18]
**Rt week 5**	0.88	[18]
**Rt week 6**	0.86	[18]
**Rt week 7**	0.84	[18]
**Rt week 8**	0.75	[18]
**Rt weeks 9–10**	0.7	Estimate
**Rt weeks 11–12**	0.65	Estimate
**Rt week 13**	0.6	Estimate
**Rt week 14**	0.56	Estimate
**Rt week 15**	0.5	Estimate
**Rt week 16**	0.46	Estimate
**Rt weeks 17–20**	0.4	Estimate
*Risk Reduction ICU (%)	56.4	[5]
*Risk Reduction Hospitalization (%)	73.5	[5]

**Table 2:** Transition probabilities. Probabilities of transitioning among patients’ states.

Hospitalization (%)	3.8	[12]
**Intensive Care Units (%)**	1.06	[14]
**Mortality (%)**	2.40	[15]
**Eligibility–cas&im (%)**	4.76	[7]

*The reduction of the risk of hospitalization was evaluated instead of the reduction of the risk of death to obtain more conservative results. Indeed, cas&im are administered to outpatients, and their effectiveness is expressed in the transition of the state of hospitalization. It is also assumed that death occurs only as a consequence of hospitalization.

The model under investigation is dynamic, implying that infected people, new patients treated with cas&im (according to the eligibility rate), new hospitalizations, new accesses in ICU, and deaths are added to the model at the end of each cycle (week 1). The starting number of infected patients (week 1) is determined from historical data and is combined over the 20 weeks’ time frame, adding the estimated number of new infected each week. The estimation of ICUs and deaths follows a similar procedure, the number of infected people and the transition probabilities are assessed from one state to the other.

### 2.4 Economic data

The perspective of the Italian National Health Service (NHS) was considered in the economic evaluation because the costs are directly associated with the length of stay in the ward and intensive care. Consequently, an increase in one of the parameters directly affects the costs, while a reduction generates savings for the hospitals. The average length of hospitalization days stems from the study by Weinreich et al. [5], while the average length in intensive care was provided by Nuovo Ospedale dei Castelli [19]. The duration of treatment comes from the study by Ash et al. [13]. The data on hospitalization and intensive care costs derive from the State General Accounting Department [17] and ALTEMS [16]. The costs of the SoC were calculated by applying the average daily tariff for hospitalization in ward to the length of stay as estimated by the National General Accounting Department [17]. In October 2021, the price of the cas&im treatment has not yet been officially made public on the Italian territory, for this reason, the US price equal to USD 2,100, or EUR 1,820, was used as a reference [20]. Details about economic data are shown in Table 3.

**Table 3:** Economic data. Economic data used to populate the model.

Parameters	Value	Source
**Hospitalization (days)**	5.5	[5]
**Intensive care unit (days)**	10	[19]
**Treatment (minutes)**	62.7	[13]
**Hospitalization Cost–standard**	674.00 €	[17]
**Intensive care unit Cost—standard**	1,654.00 €	[16]
**Treatment Cost**	1,820.00 €	[20]

### 2.5 Sensitivity analysis

This analysis determines the variation of one or more parameters on the results of the model by focusing on the parameters most sensitive to the onset of a variation.

Based on the nature of the parameters, three distributions have been assigned to each parameter:

1. *Gamma Distribution*: applied to the length of stay in the ward, intensive care, and death
2. *Beta Distribution*: applied to rates of hospitalization, intensive care, mortality, eligibility for treatment, reduction of the risk of hospitalization, reduction of the risk of admission to ICU, and reduction of the risk of death
3. *Deterministic Distribution*: applied to hospitalization, intensive care, and treatment costs.

Details on alpha and beta parameters are shown in S1 Fig.

## 3. Results

A summary of the results from the first phase of the simulation is reported in Table 4. The first phase of the model, standard treatment, estimates that within the observed time span of 20 weeks there are 194,541 positive cases for Covid-19, 7,389 hospitalizations, 3,978 admissions to ICU, and 4,658 deaths. The second phase of the model, cas&im treatment, estimates a reduction of 4,167 in the number of hospitalizations, 3,589 in intensive care, and 1,535 in deaths.

**Table 4:** Epidemiology. Results of the first simulation phase.

Parameter	Standard of Care	Cas&im Treatment	Difference
*Infected*	194,451	194,451	-
*Hospitalizations*	7,389	3,222	• 4,167
*Intensive Care Units*	3,978	388	• 3,589
*Deaths*	4,658	3,123	• 1,535

**Fig 2:**
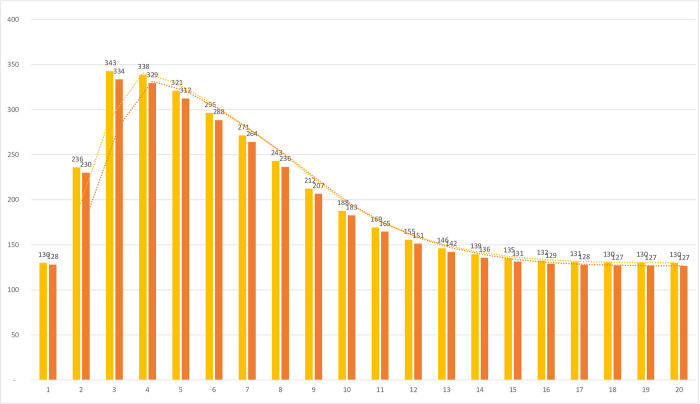
Transfers to ICU– 20 weeks. Yellow bars: standard of care. Orange bars: cas&im treatment.

Fig 2 displays a graphical representation of the ICU accesses differentiated by the type of treatment over the period of 20 weeks. The graph shows the difference in the number of accesses in intensive care when the standard of care is used (yellow bars) and when the cas&im treatment is assumed (orange bars). Considering that only 4.76% of the Italian population is eligible for cas&im treatment, the results are still promising as a significant reduction in access to intensive care is observed, which confirms the effectiveness of the treatment.

Table 5 displays the results obtained by the model in the second phase of the simulation in terms of costs for the hospital facilities according to the two treatment options. The administration of the monoclonal antibody treatment (cas&im) can generate savings for a total of EUR 78 million for the facilities concerned.

**Table 5:** Costs. Results of the second simulation phase.

Parameter	Standard of Care	cas&im Treatment	Difference
*Hospitalization costs*	€ 27,391,539	€ 11,955,597.45	- € 15,435,941.06
*Intensive care unit costs*	€ 65,801,104	€ 6,426,331.76	- € 59,374,722.42
*Death costs*	€ 61,636,839	€ 41,326,859.53	- € 20,309,979.47
***Treatment costs***	0	€ 16,845,681.46	+ € 16,845,681.46
***Total Costs***	**€ 154,829,482**	**€ 76,554,470.19**	**- € 78,275,011.49**

As it is shown in Table 5, the savings generated by the administration of cas&im stem from a reduction in the number of hospitalizations (- EUR 15 million) and access to intensive care (- EUR 59 million), causing a significant reduction in the number of deaths which generates associated savings of more than EUR 20 million.

### 3.1 Multivariate sensitivity analysis

**Fig 3:**
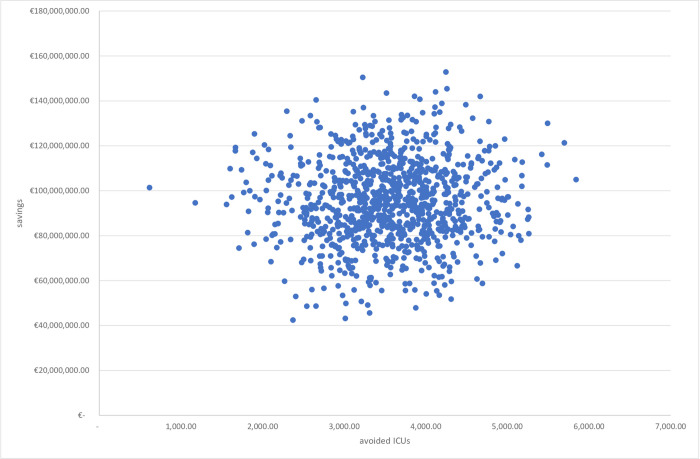
CE plane. Relationship between incremental costs and incremental ICUs.

**Fig 4:**
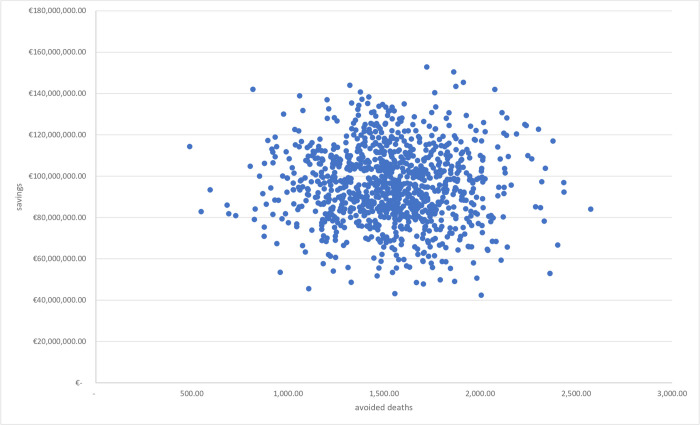
CE plane. Relationship between incremental costs and incremental deaths.

The results of the multivariate sensitivity analysis are reported in Figs 3 and 4 with two different cost-effectiveness planes. Fig 3 displays the relationship between incremental costs and incremental intensive care units. A reduction in access to intensive care generates a significant reduction in costs and, consequently, greater savings. Similarly, Fig 4 points out the relationship between incremental costs and incremental deaths. A reduction in the number of deaths translates into greater savings.

In summary, Figs 3 and 4 demonstrate that in more than 90% of the simulations the reduction in intensive care and deaths leads to greater savings, confirming the effectiveness of cas&im treatment.

### 3.2 Univariate sensitivity analysis

**Fig 5:**
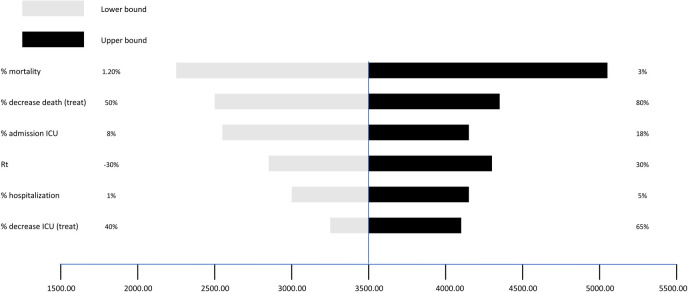
Avoided ICUs. Results of the univariate sensitivity analysis focused on ICUs.

**Fig 6:**
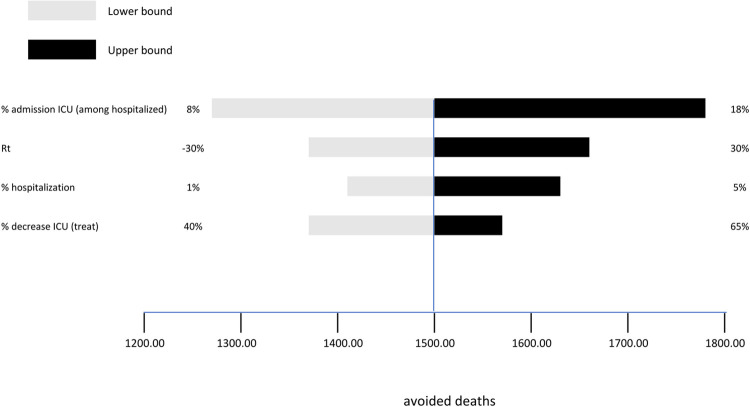
Avoided deaths. Results of the univariate sensitivity analysis on avoided deaths.

**Fig 7:**
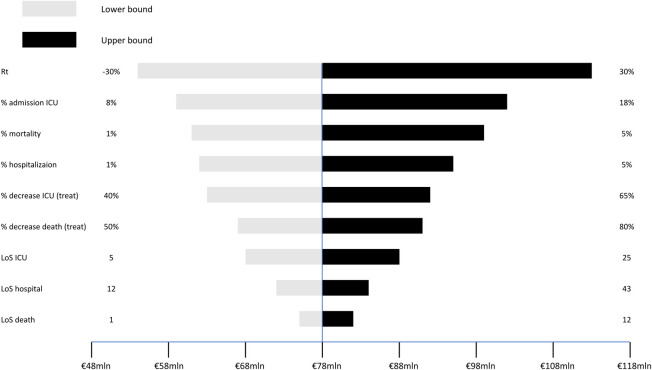
Savings. Results of the univariate sensitivity analysis on savings.

Figs 5–7 show the results of the univariate sensitivity analysis in the form of a tornado diagram. The three *Figures* point out how intensive care, deaths, and costs vary according to individual parameters included in the analysis. The gray bars correspond to the variation of the parameter of interest with the associated minimum value. The black bars show the variation of the parameter of interest with the associated maximum value. The analysis shows that the most sensitive parameters are:

- Rt
- Mortality (%)
- Admissions to Intensive Care (%)
- Hospitalization (%)
- Reduction of access to Intensive Care (%) with cas&im administration
- Reduction (%) of the number of deaths

The tornado diagram in Fig 5 shows the number of intensive care units that can be avoided. The mortality rate varies from 1.2% to 3%. Admissions to ICU associated with mortality vary from 2,300 to 5,000 patients, with a reduction of 2,700 patients. The percentage reduction in deaths varies between 50% and 80%, associated with 2,500 and 4,350 visits to intensive care, with a reduction of 1,850 patients. The rate of ICU admissions ranges from 8% to 18% with 2,550 and 4,150 admissions to ICU, with a reduction of 1,600 patients. The Rt index has a range of ±30% and its variation leads to between 2,850 and 4,300 new admissions to ICU, with a reduction of 1,450 patients. The percentage range of hospitalization varies between 1% and 5%, which is associated with 3,000 and 4,150 accesses to intensive care, with a reduction of 1,150 patients. The percentage reduction in ICUs due to the treatment varies between 40% and 65% with 3,250 and 4,100 patients in ICU, with a reduction of 850 patients.

The tornado diagram in Fig 6 focuses on the deaths that can be avoided. The rate of admissions to intensive care varies from 8% to 18% with, respectively, 1,270 and 1,760 deaths, with a reduction of 490 patients. The Rt index varies between ±30% with 1,360 and 1,660 deaths, with a reduction of 300 patients. The hospitalization rate varies between 1% and 5% with 1,410 and 1,630 deaths, with a reduction of 220 patients. The percentage reduction of access to intensive care thanks to the cas&im treatment varies between 40% and 65% with 1,360 and 1,570 deaths, with a reduction of 210 patients.

The tornado diagram in Fig 7 focuses on the savings that can be generated from each individual variable. The contagion index Rt, which varies between ±30%, is associated with costs that amount to EUR 53 million and EUR 112 million, with cost savings of EUR 59 million. The percentage of admission to intensive care varies between 8% and 18% with associated costs between EUR 58 million and EUR 101 million, with cost savings of EUR 43 million. The mortality rate, which varies between 1% and 5%, generates costs between EUR 61 million and EUR 98 million, with cost savings of EUR 37 million. The percentage of hospitalization varies between 1% and 5% with EUR 62 million and EUR 95 million of associated costs, with cost savings of EUR 33 million. The percentage reduction in ICU admissions due to the cas&im treatment varies between 40% and 65% with associated costs between EUR 63 million and EUR 92 million, with cost savings of EUR 29 million. The percentage reduction in deaths thanks to cas&im treatment varies between 50% and 80% with associated costs between EUR 67 million and EUR 91 million, with cost savings of EUR 24 million. The length of stay in ICU varies between 5 days and 25 days with associated costs of EUR 68 million and EUR 89 million, with cost savings of EUR 21 million. The length of stay in ward varies between 12 days and 43 days with associated costs that amount to EUR 72 million and EUR 84 million, with cost savings of EUR 12 million. The length of stay to death varies between 1 and 12 days with associated costs of EUR 75 million and EUR 82 million, with cost savings of EUR 7 million.

## 4. Discussion

The results of this study confirm the efficacy of cas&im from a healthcare and economic point of view in the treatment of outpatients with Covid-19 as already found in other studies [5, 6, 13, 21–24]. A comparison with other similar studies is not possible as there are no cost-effectiveness analyses for outpatients treated with cas&im for Covid-19. However, an academic study [25], which is limited to the ambulatory setting, confirms that cas&im treatment is cost-effective in most patients.

The main advantage of this treatment is that, compared to other therapeutic solutions, it acts at the root of the problem, producing a favorable chain reaction and a more positive outcome for the patient with COVID-19. It is estimated that the administration of cas&im can reduce the number of hospitalizations (- 4,167), the number of intensive care units (- 3,589), and deaths (- 1,535), producing cost savings of more than EUR 78 million in the period of 20 weeks. The cost savings are also made possible by the rapidity of the administration of the treatment, which is approximately one hour, compared to other treatments that require several days of hospitalization. From a hospital management point of view, the risk of crowding in the wards is significantly reduced, as is the use of intensive care.

This study has several limitations. First, the predictive epidemiological model is based on estimated parameters, such as the contagion index Rt. Then, the economic model does not employ the real price of the treatment as it has not yet been made public, this analysis is carried out using the US price which may not perfectly correspond to the actual price of the treatment in Italy. Further, academic studies on the variability of hospitalization and the stay in intensive care when cas&im are administered are missing, which are fundamental in the economic analysis. In addition, the main advantages of cas&im may emerge only if the therapy is administered on a preventive level, i.e., on individuals who have been exposed to COVID-19 patients in different circumstances and have no symptoms yet. At present, these advantages cannot be estimated because of the low eligibility rate for cas&im administration. Also, results by QALYs were not presented because there are no utility coefficients. Indeed, the population is heterogeneous and no distribution is available according to age or comorbidity. In conclusion, the 4.76% eligibility [7] that was employed in this investigation was determined when the alpha (B.1.1.7) and delta (B.1.617.2) variants were prevalent, while at the moment the omicron variant (B.1.1.529 and, specifically, its subvariant BA.5) is the most frequent worldwide. The data show that cas&im is not likely to be active against the omicron variant [26].

The strengths of this study are manifold. Thanks to the adaptability of the model, problems in the lack of substantial data will be solved as the data is made available. Furthermore, the model can effectively guide decision-making activities, mainly during a state of emergency. The model has also the ability to make estimates at a more limited territorial level, such as regions, which have diversified organizational and logistical healthcare structures. From a preventive future perspective, the model can simulate the pandemic course resulting from viruses other than SARS-CoV-2, applying appropriate adaptations.

### 5. Conclusion

This study shows the benefits that the use of cas&im treatment can generate for healthcare facilities and patients. Hospitals can achieve important cost savings thanks to better use of scarce resources such as hospitalizations and intensive care, while patients can experience a more favorable disease course in terms of reduction of hospitalization days, transfer to intensive care, and death.

## Supporting information

S1 Fig Alpha and Beta parameters. The figure shows alpha and beta values for each parameter.
(TIF)
