# Supplementary figures and images for: Casirivimab and imdevimab: Cost-effectiveness analysis of the treatment based on monoclonal antibodies on outpatients with Covid-19
Source: PLoS One. 2023 Feb 10;18(2):e0279022. doi: 10.1371/journal.pone.0279022 (PMC9916561; doi:10.1371/journal.pone.0279022)

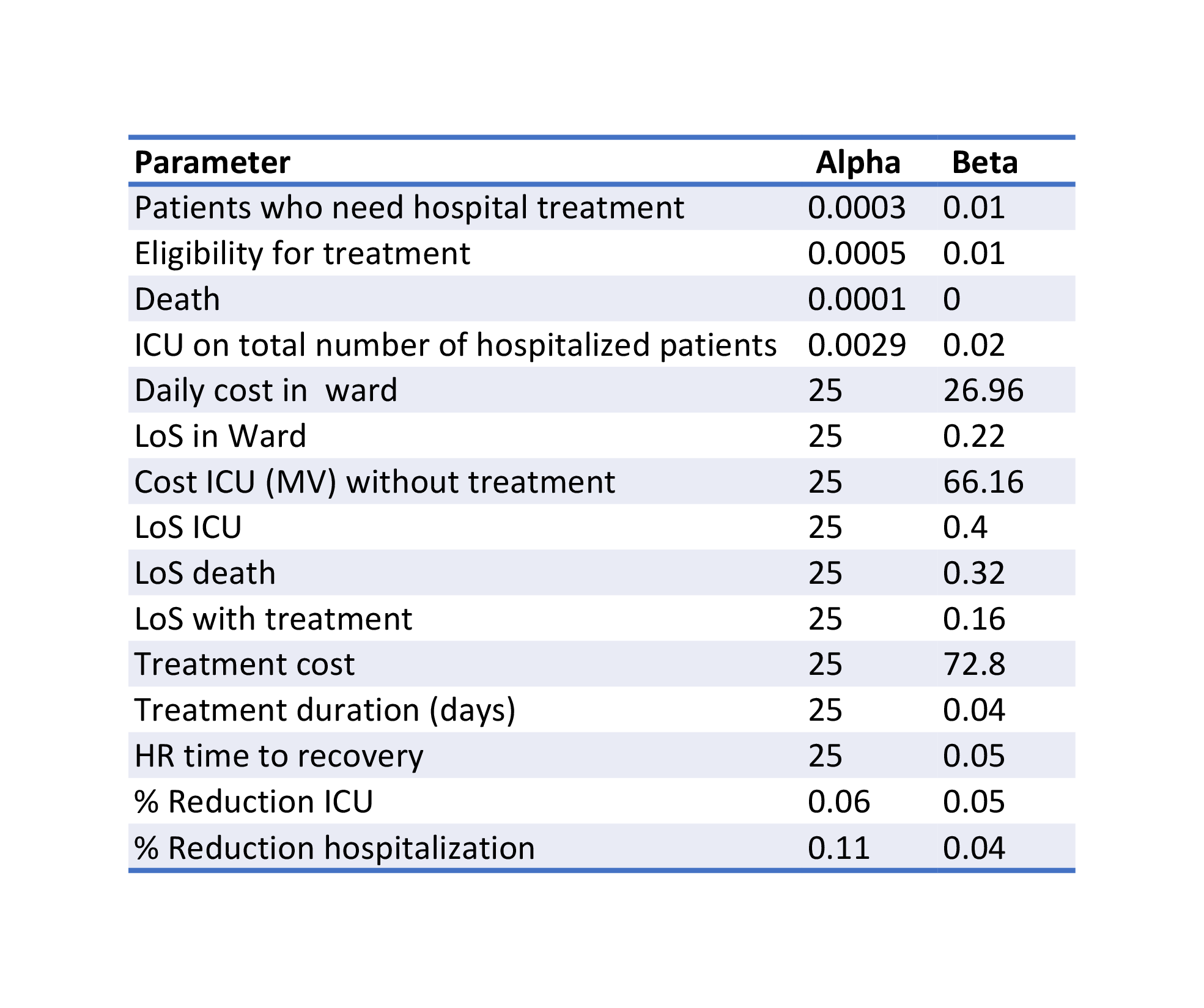

Supplement: S1 Fig — The figure shows alpha and beta values for each parameter. (TIF) [file pone.0279022.s001.tif]
